# Reverse Signaling by MHC-I Molecules in Immune and Non-Immune Cell Types

**DOI:** 10.3389/fimmu.2020.605958

**Published:** 2020-12-15

**Authors:** Elke M. Muntjewerff, Luca D. Meesters, Geert van den Bogaart, Natalia H. Revelo

**Affiliations:** ^1^ Department of Tumor Immunology, Radboud Institute for Molecular Life Sciences, Radboud University Medical Center, Nijmegen, Netherlands; ^2^ Department of Molecular Microbiology and Immunology, Groningen Biomolecular Sciences and Biotechnology Institute, University of Groningen, Groningen, Netherlands

**Keywords:** reverse MHC class I signaling, immunological synapse, cell activation, proliferation, apoptosis, migration, dendritic cell, tumor immune responses

## Abstract

Major histocompatibility complex (MHC) molecules are well-known for their role in antigen (cross-) presentation, thereby functioning as key players in the communication between immune cells, for example dendritic cells (DCs) and T cells, or immune cells and their targets, such as T cells and virus-infected or tumor cells. However, much less appreciated is the fact that MHC molecules can also act as signaling receptors. In this process, here referred to as reverse MHC class I (MHC-I) signaling, ligation of MHC molecules can lead to signal-transduction and cell regulatory effects in the antigen presenting cell. In the case of MHC-I, reverse signaling can have several outcomes, including apoptosis, migration, induced or reduced proliferation and cytotoxicity towards target cells. Here, we provide an overview of studies showing the signaling pathways and cell outcomes upon MHC-I stimulation in various immune and non-immune cells. Signaling molecules like RAC-alpha serine/threonine-protein kinase (Akt1), extracellular signal-regulated kinases 1/2 (ERK1/2), and nuclear factor-*κ*B (NF-*κ*B) were common signaling molecules activated upon MHC-I ligation in multiple cell types. For endothelial and smooth muscle cells, the *in vivo* relevance of reverse MHC-I signaling has been established, namely in the context of adverse effects after tissue transplantation. For other cell types, the role of reverse MHC-I signaling is less clear, since aspects like the *in vivo* relevance, natural MHC-I ligands and the extended downstream pathways are not fully known.The existing evidence, however, suggests that reverse MHC-I signaling is involved in the regulation of the defense against bacterial and viral infections and against malignancies. Thereby, reverse MHC-I signaling is a potential target for therapies against viral and bacterial infections, cancer immunotherapies and management of organ transplantation outcomes.

## Introduction

Major histocompatibility complex class I (MHC-I) molecules are present on all nucleated cells and are classically known for presenting peptides derived from endogenous antigens to cytotoxic CD8^+^ T lymphocytes (CTL). Additionally, in the case of professional antigen presenting cells (APCs) [*i.e.* dendritic cells (DCs), macrophages and B cells], MHC-I can also present exogenous antigens through a process called antigen cross-presentation. Moreover, MHC-I molecules are important immune regulators, since their expression levels regulate activation of natural killer (NK) cells (1, 2).

An underappreciated function of MHC-I molecules is their ability to act as signaling receptors. In this process, here referred to as reverse MHC-I signaling, ligation of MHC molecules can lead to signal-transduction and cell regulatory effects in the APC (3, 4). Multiple studies have shown that reverse MHC-I signaling can influence processes like cell activation, proliferation, maturation, cytotoxicity, and migration, or even lead to cell anergy and apoptosis (3, 5, 6). MHC-I reverse signaling has been observed in multiple cell types, ranging from immune cells, such as macrophages, NK cells, T cells, and B cells, to non-immune cells like endothelial and smooth muscle cells (7, 8). Furthermore, reverse MHC-I signaling has been investigated in the context of viral and bacterial infections (6, 9), transplantation immunity (10), malignancies (11), and brain development (12). Here, we review the evidence for the alternative role of MHC-I as reverse signaling molecules across immune and non-immune cells.

### MHC-I Function in T Cell Activation and NK Cell Regulation

MHC molecules [referred to as human leukocyte antigen (HLA) in humans and histocompatibility system 2 (H-2) in mice] play an important role in the communication between the innate and adaptive immune system. There are two classes of MHC molecules that are involved in antigen presentation: MHC class I and MHC class II. MHC-I molecules are present on all nucleated cells and classically function to activate CTLs with endogenous antigens, whereas MHC-II molecules are present on professional antigen-presenting cells and are involved in the activation of CD4^+^ T cells with exogenous antigens (1). Exogenous antigens can also be presented by MHC-I to CD8^+^ T cells, in a process called antigen cross-presentation, which is important for the defense against tumors and intracellular pathogens (13).

**Figure 1:**
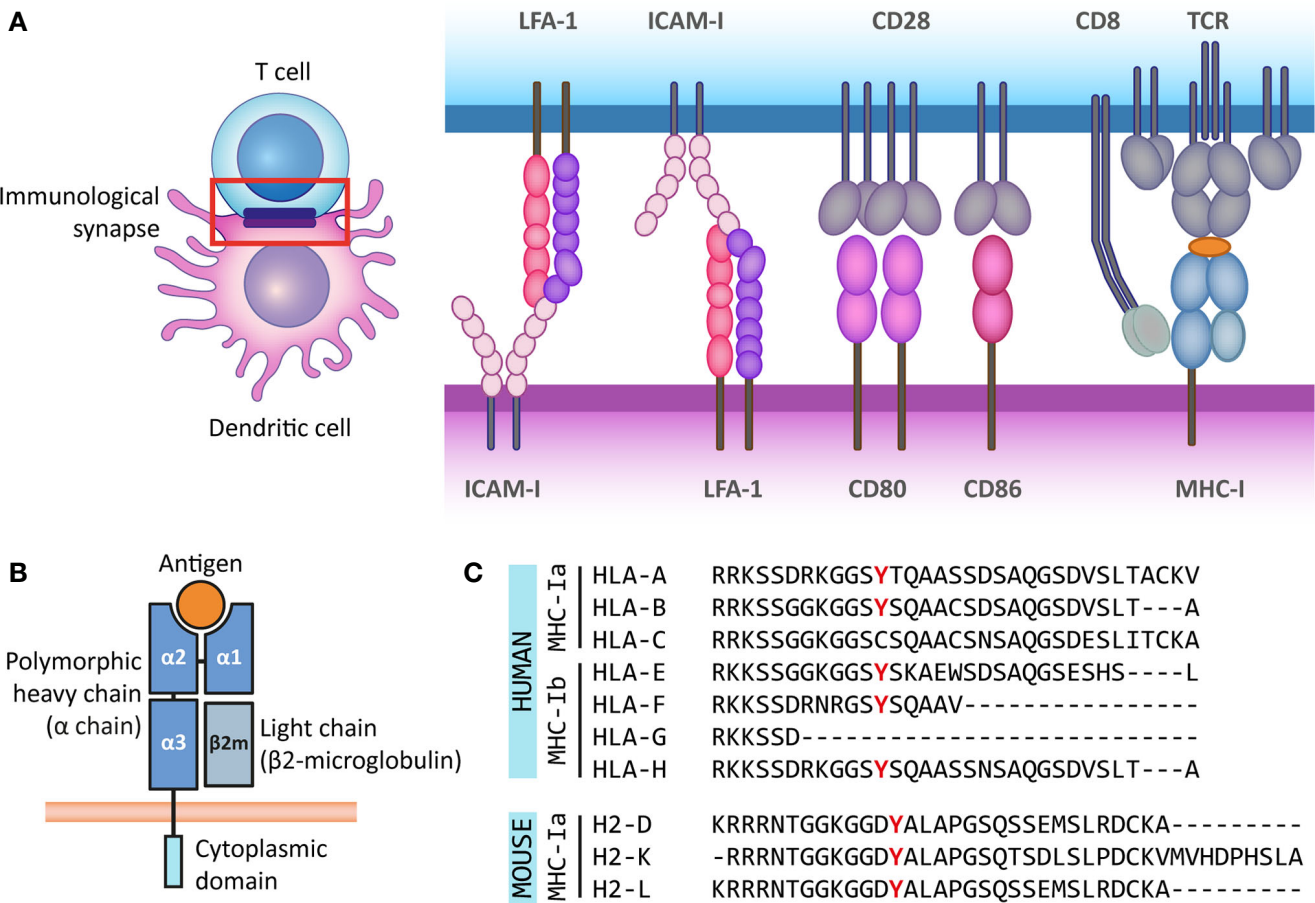
The antigen presenting role of MHC class I molecules and their structure. **(A)** The immunological synapse is a communication platform between a (professional) antigen-presenting cell and a T or NK cell. In the example presented here, MHC-I molecules on the surface of a dendritic cell (purple) present antigens (orange) for their recognition by the TCR of a CD8^+^ T cell (blue). CD8 acts as co-receptor of the TCR for the recognition of the MHC-I molecule. At the immunological synapse (red box), other molecules required for the engagement and modulation of the interaction are also recruited, such as adhesion molecules (LFA-1 and ICAM-I) and costimulatory molecules (CD80/86 and CD28). **(B)** MHC-I molecules are heterodimers composed of a heavy *α* chain (blue) and a light chain (*β*2-microglobulin) (gray). Most *α* chains are made up of two peptide binding domains (*α*1 and *α*2), and an Ig-like *α*3 domain recognized by CD8 molecules on T cells. Additionally, the *α* chain has a transmembrane and a short cytoplasmic tail (cyan). **(C)** The cytoplasmic regions of several classical and non-classical MHC-I *α* chains contain a tyrosine residue that can be phosphorylated. This phospho-tyrosine residue has been proposed to interact with signaling proteins and thus leads to reverse signaling. Here we present the cytoplasmic regions of human classical (MHC-Ia) and non-classical (MHC-Ib) *α* chains, and the classical mouse *α* chains. However, some of the multiple non-classical mouse *α* chains also contain this tyrosine residue.

The interface formed between an APC and an antigen-recognizing T cell is called an immunological synapse. T cell activation requires the delivery of three molecular signals by the APC: First, the complex formed by MHC and the antigenic peptide is recognized by the T cell receptor (TCR) along with the co-receptor CD4 on helper T cells (for MHC-II) or CD8 on CTLs (for MHC-I). Second, a costimulatory signal provided by CD80/86 is recognized by CD28 on the T cell. Third, the APC releases stimulatory cytokines that are recognized by their receptors on the T cell. Additionally, the immunological synapse is stabilized by adhesion molecules such as lymphocyte function-associated antigen (LFA)-1 and intercellular adhesion molecule 1 (ICAM-1). The combination of costimulatory signals and cytokines secreted by the APC determines the functional outcome of the interaction, such as the activation or inhibition of the T cell (14) (**Figure 1A**). The immunological synapse can be seen as a radially symmetric structure consisting of several supramolecular activation clusters (SMACs) (15). The classical synapse contains a central SMAC (cSMAC), where the MHC-TCR interactions, co-receptors (CD3 and CD4 or CD8) and costimulatory molecules are located. The surrounding peripheral SMAC (pSMAC) includes adhesion molecules LFA-1 and ICAM-1, and the distal SMAC (dSMAC) contains the transmembrane tyrosine phosphatase CD45, which activates signaling molecules promoting the T cell response (14, 16, 17). There are exceptions to this structure, for instance when T cells interact with DCs, where a multifocal synapse is formed (16, 18).

In addition to their role in CD8^+^ T cell activation, MHC-I molecules provide signals that are crucial for the regulation of different immune cells. For instance, NK cells carry inhibitory receptors that bind to MHC-I on the surface of other cells, independent of the antigen presented, thereby suppressing NK cell activation. This is particularly important as viruses and malignancies often downregulate MHC-I expression to circumvent T cell detection, requiring the action of NK cytotoxic function (2). In mice, one type of inhibitory receptor is the Ly49 family, which has a C-type lectin-like structure, with two transmembrane domains. Their functional homologs in human are the killer Ig-like receptors (KIRs), which belong to the Ig-superfamily. These two families have similar inhibitory functions despite their differences in structure and evolutionary origin. Additionally, in both species the C-type lectin-like molecules CD94 and NKG2 (CD94/NKG2) form heterodimers with inhibitory properties (2). Another important group of MHC-I receptors are the Leukocyte Immunoglobulin-Like Receptors (LILRs), also known as CD85. They can have both activating and inhibitory roles and have been found in immune (*e.g.* lymphocytes, neutrophils, eosinophils, macrophage, DCs) and non-immune cells (*e.g.* endothelial cells) (19).

### The Alternative Role of MHC-I as Signaling Molecules and Their Structure

Besides their role in antigen presentation for T cell activation, both MHC-I and MHC-II molecules are involved in reverse signaling, meaning that ligation of the MHC molecule leads to signal-transduction and cell regulatory effects in the APC (3). In this review we will focus on reverse MHC-I signaling; given the ample literature available across different cell types, its importance for medical outcomes in transplantation patients and its potential role regulating immune responses against infection and malignancies.

So far, there is no consensus on the molecular mechanism underlying reverse MHC-I signaling, as the existing hypotheses rely on different domains of the complex for signal transduction or different MHC-I subclasses. MHC-I molecules are heterodimers composed of a heavy *α*-chain and a light chain called beta-2 microglobulin (*β*_2_m). Typically, the *α*-chain contains domains *α*1 and *α*2, both involved in peptide binding, and the immunoglobulin-like *α*3 domain, proximal to the membrane, which is recognized by CD8 co-receptors on the T cell (1). *β*_2_m expression is important for the stable expression of the *α*-chain at the cell surface, the proper binding of the complex to the antigenic peptide for presentation and for TCR recognition (20, 21). The transmembrane helix of the *α*-chain anchors the MHC-I complex to the membrane. The *α*-chain also has a short cytoplasmic tail of around 30 to 40 amino acids (**Figure 1B**).

Multiple genes, grouped into MHC gene clusters, encode for heavy *α*-chains in mouse and human, and are divided in classical and non-classical MHC molecules. The classical MHC-I molecules (MHC-Ia) are responsible for activating CD8^+^ T cells and correspond to *α*-chains that are expressed at high levels and are highly polymorphic to allow for increased variability of peptide binding sites. In humans, these correspond to the HLA-A, HLA-B, and HLA-C genes, and in mice, these genes are H-2K, H-2D and H-2L. The non-classical MHC-I molecules (MHC-Ib) are expressed at lower levels and are less polymorphic. They participate in activation or regulation of NK cells, among other less understood functions. In humans MHC-Ib molecules include genes HLA-E, HLA-F, HLA-G, and the pseudogene HLA-H, whereas in mice it includes several genes under the H2-Q, H2-T, and H2-M regions (22–25). Interestingly, the cytoplasmic domains of some classical and non-classical *α*-chains in different species have a conserved tyrosine residue (9, 26) (**Figure 1C**). As discussed in the following sections, part of the evidence for reverse MHC-I signaling is supported by the notion that this tyrosine residue can be phosphorylated and that tyrosine phosphorylation is a posttranslational modification frequently involved in signal transduction (27). Besides this, based on their structure, MHC-I molecules belong to the Immunoglobulin (Ig) superfamily, which is widely involved in cellular recognition and intercellular signaling (6, 28). Moreover, the existence of open conformers, cell surface MHC molecules that are not associated with peptide and/or *β*_2_-microglobulin (*β*_2_m), and their ability to associate with themselves or other receptors, suggest that MHC-I molecules have more functions than only antigen presentation to T and NK cells (29). The formation of open conformers is also associated with tyrosine phosphorylation, further supporting the idea of reverse signaling by MHC-I (30).

## Reverse MHC-I Signal Transduction in Myeloid Immune Cells

### Macrophage Activity Regulation by Reverse MHC-I Signaling

Early evidence of MHC-I reverse signaling in macrophages was already reported in 1987 (**Table 1**). Immunoglobulin G2 (IgG2) antibodies that targeted MHC-I, but not the corresponding F(Ab′)_2_ fragments, induced guinea pig alveolar macrophages to release reactive oxygen species (ROS) in the oxidative burst (31). This increase was not observed for IgG1 antibodies, which even reduced ROS release, whereas their F(Ab′)_2_ did not have an effect (31). This finding was explained as a consequence of bipolar bridging, an event in which an antibody binds simultaneously to an Fc-receptor *via* its Fc-tail and to MHC-I with its antigen-binding Fab motif on the same membrane (*cis* bridging). Therefore, this finding cannot be considered as solely MHC-I reverse signaling, but as an effect of collaboration between MHC-I and the Fc receptors for IgG2 or IgG1. Notably, although the antibody Fc-tail could also bind to an Fc receptor on a different cell (*trans* bridging), different authors claim that bipolar bridging is a monocellular event (31, 33). The main evidence for this comes from experiments with mast cell mixtures where only those cells expressing both the right H-2 antigen and Fc-receptor degranulate upon antibody binding (34), and with antibody-induced degranulation of single cells isolated with a micromanipulator (35) (see also section *Reverse MHC-I Signaling in Peritoneal Cells Induces Mast Cell Degranulation*). Furthermore, the bipolar bridging hypothesis relies on physicochemical models proposing that the high local concentration of proteins at the plasma membrane makes it easier for an antibody to bind to MHC-I and Fc-receptors on the same cell, rather than on two different cells (36, 37). The physiological relevance of simultaneous signaling during bipolar bridging is unclear, because it is unknown when for instance a CD8^+^ T cell or an NK cell would display ligands for both MHC-I and Fc-receptors.

**Table 1:** Summary MHC-I signaling in macrophages and dendritic cells.

Cell type	MHC-I stimulation	Signaling	Cell outcome	Ref
Guinea pig alveolar macrophages	Guinea pig IgG1 and -2 antibodies	–	In combination with Fc-receptor stimulation, ↑ or ↓ release of oxygen free radicals	(31)
Mouse peritoneal macrophages	TLR ligands, bacteria, monoclonal antibodies (anti-H-2Kb; AF6-88.5)	↑ Fps, SHP-2 ↓TRAF6, NF-κB	↓ TLR response	(9)
Mouse bone marrow-derived macrophages	VSV infection, IFN-β	↑ SHP2 ↓STAT1, ISG	↓ type I IFN signaling and ↑ viral infection	(6)
Human monocyte-derived dendritic cells	Synapse with CD8^+^ T cells or beads coated with mouse anti HLA-A2 antibodies	–	Remodeling of recycling endosomes from vesicular to tubular structures	(32)
Mouse bone marrow-derived dendritic cells	β2-microglobulin knock out mice	–	Increased production of TNF, IL-6 and IFN-β after TLR stimulation	(9)

**Figure 2:**
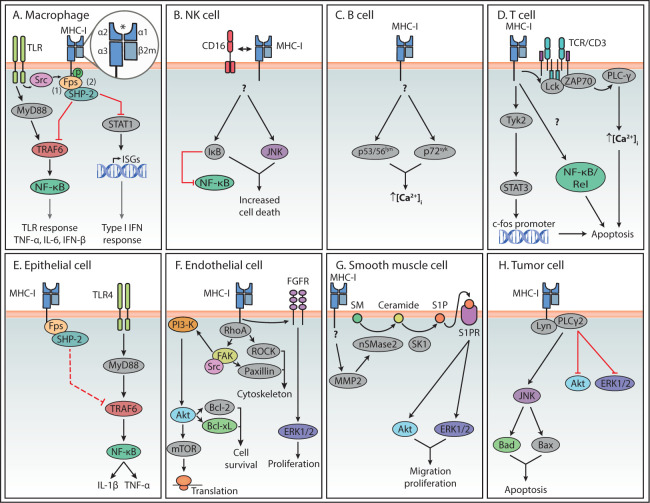
Summary of reverse MHC-I signaling pathways in various cell types. Summary of the main reverse MHC-I signaling pathways in immune **(A–D)** and non-immune cells **(E–H)**. Question marks indicate steps in the pathway that are still unknown. Interactions represented by solid lines were confirmed to participate in reverse MHC-I signaling, while the interaction represented with a dashed line in (E) represents the authors**’** speculation. The asterisk symbol (*) indicates the antigen binding site of MHC-I. In the case of Endothelial cells **(F)**, reverse MHC-I signaling has been well studied and only a brief overview is presented here. A more detailed description can be found in a review by Valenzuela and Reed, 2011 (5).

A few decades later, a modulatory effect by MHC-I reverse signaling on the toll-like receptor (TLR) signaling cascade was described. When compared to *β*_2_m knock-out mice, it was established that normal expression of MHC-I suppresses the TLR response, including the production of tumor necrosis factor (TNF), interleukin (IL)-6 and interferon-beta (IFN-*β*). This TLR suppression is suggested to restrict innate inflammatory responses upon bacterial infection, to prevent excessive damage as seen during septic shock (9). However, this data should be interpreted with caution since *β*_2_m also dimerizes with the heavy chains of other MHC-I-like surface molecules such as CD1 and the neonatal Fc-receptor (FcRn), and therefore its deletion might have effects beyond MHC-I function (20). In mouse peritoneal macrophages, this MHC-I-induced inhibition of TLR-mediated inflammatory responses was shown to be induced by interaction with naïve CD8^+^ T cells (9). Based on experiments using MHC class I ligation with antibodies, this study proposed a mechanism in which the Src homology 2 (SH2) domain of the tyrosine kinase Fps interacts with a phosphorylated tyrosine site in the cytoplasmic domain of MHC-I, leading to Fps activation (**Figure 2**) (9). TLR stimulation activates the kinase Src, which was suggested to phosphorylate the tyrosine in the cytoplasmic domain of MHC-I, thus facilitating its association with Fps (9). In turn, activated Fps associates with the phosphatase SHP-2, which then interacts with TNF receptor associated factor (TRAF) 6, thereby downregulating its ubiquitination and activity. In this way, MHC-I stimulation suppresses the MyD88-TRAF6-mediated activation of NF-*κ*B and thus inhibits TLR downstream responses (9).

In addition to the role of MHC-I reverse signaling in dampening TLR signaling, its roles in type I interferon (IFN) signaling and antiviral infections has been investigated. Using bone marrow-derived macrophages from MHC-I-deficient mice, it was shown that MHC-I promotes replication of vesicular stomatitis virus (VSV) in macrophages independent of T cells (6). Additionally, MHC-I plays a regulatory role in type I IFN signaling, which was shown by increased phosphorylation of signal transducer and activator of transcription 1 (STAT1) and elevated interferon-stimulated gene (ISG) expression in MHC-I-deficient macrophages (6). To fulfill this function, MHC-I depends on phosphorylation of its intracellular tyrosine site, as shown by immunoprecipitation. Moreover, SHP-2 was necessary for suppression of IFN signaling by MHC-I engagement, *via* interaction of SHP-2 with STAT1 after VSV infection (6).

In conclusion, MHC-I seems to be involved in reverse signaling in macrophages, either alone or in collaboration with Fc-receptors, thereby affecting ROS release, TLR responses and type I IFN responses. Therefore, reverse MHC-I signaling may regulate the activity of macrophages to prevent an excessive response and damage (9). This effect could be induced after interaction with the TCR on CD8^+^ T cells (9), but potentially also receptors on NK-cells, such as KIR receptors or C-type lectin-like receptors like Ly49 (38).

### Reverse MHC-I Signaling Modulates Membrane Trafficking and Cytokine Production in Dendritic Cells

In comparison to the other cell types discussed in this review, reverse MHC-I signaling has been understudied in DCs (**Table 1**). In human monocyte-derived DCs (moDCs) it was demonstrated that establishment of antigen-specific immunological synapses with CD8^+^ T cells induces the remodeling of endosomal recycling compartments (ERC), going from a vesicular to a tubular morphology (32). To dissect the molecular mechanisms of ERC remodeling, moDCs were stimulated with antibody-coated beads. These experiments revealed that ERC remodeling could be triggered by beads coated with antibodies against MHC-I (HLA-A2) and/or antibodies against ICAM-I molecules. Furthermore, remodeling was more extensive in moDCs previously activated with LPS than in immature moDCs. The tubules were suggested to facilitate the transport of antigen-loaded MHC-I molecules to the immunological synapse. However, this study did not explore the signaling pathway(s) triggered by MHC-I stimulation that lead to endosomal remodeling (32).

In the same study reporting the modulation of TLR downstream signaling by reverse MHC-I signaling in macrophages (9) (see section *Macrophage Activity Regulation by Reverse MHC-I Signaling*), it was shown that bone marrow-derived DCs (BMDCs) derived from mice lacking *β*2-microglobulin release higher amounts of TNF, IL-6, and IFN-*β* than their wild-type counterparts upon TLR stimulation. These results suggest that, similar to macrophages, MHC-I might have a regulatory role on TLR signaling in DCs (9). However, this study only studied the mechanism of reverse MHC-I signaling in macrophages.

### Reverse MHC-I Signaling in Peritoneal Cells Induces Mast Cell Degranulation

In an early study, alloantibodies against MHC molecules were generated by immunizing mice of the CBA strain with spleen cells from mice of the A/JAX strain, which has a different alloantigen profile. When serum from the CBA immunized mice was applied to BALB/c mast cells, increased degranulation from 3% in non-treated cells to 61% in treated cells was observed (39) (**Table 2**). This physiological degranulation was attributed to alloantibodies against H-2 molecules, mainly from the IgG1 type (43). However, it cannot be concluded that this was solely the result of MHC-I reverse signaling, because this serum may have contained antibodies against other biomolecules, such as MHC-II, and thus these experiments should be repeated with purified monoclonal anti-MHC-I antibodies. Additional experiments showed that degranulation was caused by antibody bipolar bridging (see section *Macrophage Activity Regulation by Reverse MHC-I Signaling*), which is the simultaneous binding of the antibody paratopes to H-2 molecules and of the Fc tails to Fc-receptors on the same mast cell (34, 39, 40). This induced degranulation of mast cells was later confirmed with monoclonal IgG2a antibodies directed against H-2 antigens (41) and polyclonal antibodies against H-2 K and H-2 D (40). However, to be able to draw conclusions about the specific role of MHC-I reverse signaling in mast cells, anti-MHC-I antibodies need to be compared with their F(ab**’**)_2_ fragments and irrelevant antibodies, since degranulation is known to be caused by Fc-receptor signaling (44). If reverse MHC-I signaling contributes to mast cell degranulation *in vivo*, it might be involved in the inflammatory process induced by antibodies or other inflammatory cells such as CD8^+^ T cells.

**Table 2:** Summary MHC-I signaling in peritoneal cells and neutrophils.

Cell type	MHC-I stimulation	Signaling	Cell outcome	Ref
Mouse peritoneal cells	Serum of CBA mice immunized with A/JAX spleen cells	–	↑ mast cell degranulation	(39)
Mouse peritoneal cells	Anti-H-2K and anti-H2D	–	↑ mast cell degranulation	(40)
Mouse peritoneal cells	Anti-H-2 antibodies (IgG2a)	–	↑ mast cell degranulation	(41)
Peripheral and peritoneal guinea pig neutrophils	Anti-B and anti-Ia antibodies	–	↑superoxide anion $\left(O_{2}^{-}\right)$ release	(33)
Human peripheral blood neutrophils	Anti-MHC-I antibodies (W6/32)	–	↓cell death	(42)

### Neutrophil Activity and Cell Death Might Be Affected by Reverse MHC-I Signaling

As shown for guinea pig macrophages, bipolar bridging of MHC and Fc-receptors on peripheral and peritoneal guinea pig neutrophils by IgG_2_ induced superoxide anion $\left(O_{2}^{•-}\right)$ release (33) (**Table 2**). Moreover, in human peripheral blood neutrophils, ligation of MHC-I with F(ab′)_2_ fragments of the antibody W6/32 also induced signal transduction, reflected in reduced apoptosis rate (42). Furthermore, it was shown that as neutrophils start aging in culture, their apoptotic activity increases along with a reduction of MHC-I surface expression, suggesting a reciprocal relation between both phenomena. In line with this hypothesis, the *in vitro* treatment of neutrophil cultures with granulocyte-macrophage colony-stimulating factor (GM-CSF) or cyclic adenosine monophosphate (cAMP), two molecules that delay age-related apoptosis, also blocked the downregulation of MHC-I (42). Similar to mast cell degranulation, $\left(O_{2}^{•-}\right)$ release and decreased apoptosis in neutrophils by reverse MHC-I signaling might contribute to inflammation upon encountering an activated inflammatory cell or infected cell.

## Reverse MHC-I Signal Transduction in Lymphocytes

### Reverse MHC-I Signaling Affects Cytotoxicity and Cell Death of NK Cells

Early studies on the role of *β*_2_m and reverse MHC-I signaling in NK cell activity led to contradictory findings, probably because some studies used the full fraction of human peripheral blood lymphocytes or spleen leukocytes (45–48), while another study used enriched NK cells (49). While the former studies proposed that reverse MHC-I signaling in NK cells reduces their cytotoxic activity when compared to unstimulated NK cells, the latter study found the opposite (**Table 3**). An alternative reason for these differences could be the use of different antibodies to stimulate MHC-I. Evidence that this indeed might be the case, comes from another early study (50). Using NK cell lines, such as NK3.3, and primary NK cells, it was shown that different anti-MHC-I antibodies have either an stimulating, inhibiting or no effect on their lytic activity against cancer cell lines (*e.g.* leukemic K562 and lymphoblastic MOLT-4), when compared to unstimulated or irrelevant antibody (Cenox 3.53)-stimulated cells as a control (50). This variability in effects of anti-MHC-I antibodies was also observed for the induction of the oxidative burst in macrophages (31).

**Table 3:** Summary MHC-I signaling in natural killer cells.

Cell type	MHC-I stimulation	Signaling	Cell outcome	Ref
NK cell line clones and NK cell bulk populations	Anti-MHC-I antibodies (131, 164, W6/32, 404, 4E, 46)	–	↑/↓/no effect on lytic activity	(50)
Human peripheral blood NK cells	Anti- *β*_2_m antibodies (**Table 1** in article)	–	↑ lytic activity	(49)
Human peripheral blood NK cells	Anti-MHC-I antibodies (PA.2.6 HB118 IgG1)	–	In combination with CD16 stimulation, ↓ NK cell cytotoxicity ↑cell death	(51)
Human peripheral blood NK cells and NK cell line YT	Anti-MHC-I antibodies (PA.2.6 HB118 IgG1)	↑ IkB, JNK ↓ NF-*κ*B	In combination with CD16 stimulation, ↑NK cell death	(52)
Human peripheral blood NK cells and NKL cell line	Anti-MHC-I antibodies (W6/32 IgG2a) and secondary antibodies (sheep anti-mouse IgG F(ab’)_2_)	–	↓ conjugation with target and ↓ cytolytic activity	(53)
Human peripheral blood NK cells and NKL cell line	Anti-MHC-I antibodies (W6/32 IgG2a)	–	↓cytotoxicity and ↓IFN-*γ* production	(54)

More recent studies found that stimulation with anti-MHC-I antibodies inhibits cytotoxic activity of human peripheral blood NK cells and NK cell lines (51, 53, 54). Reverse MHC-I signaling was studied in the context of treatment with either anti-CD16 antibodies, which inhibited NK cell cytotoxicity, or IL-2 activation, which augmented cytotoxicity. MHC-I antibodies in combination with anti-CD16 antibodies or IL-2 treatment, further inhibited cytotoxicity of human blood NK cells against several tumor cell lines when compared to an isotype antibody control (IgG1), unstimulated cells or cells stimulated only with IL-2 or CD16 (51). Since anti-MHC-I antibodies alone did not have any effect, an interaction between MHC-I and CD16 or IL-2 receptor signaling could be needed for blocking cytotoxicity. Blocking of NF-κB with a cell permeable inhibitory peptide in CD16 antibody-treated human blood NK cells reduced cytotoxicity, suggesting a role of NF-κB in the downstream signaling pathway (52). When using the NKL cell line, derived from a human leukemia patient, cytotoxic activity against K562 cells was not affected by MHC-I ligation with the mouse monoclonal W6/32 antibody (53). However, cross-linking of MHC-I molecules by further addition of sheep-anti-mouse IgG inhibited cytotoxic activity, which was confirmed for human blood NK cells (53). Compared to ligation with only a primary monoclonal antibody, efficient cross-linking requires a secondary antibody and induces clustering and interaction between the target molecules (55), potentially leading to increased reverse MHC-I signaling. The inhibition of cytotoxicity after cross-linking was not caused by an induction of NK cell apoptosis, masking of MHC-I on the target cells, or reciprocal NK cell-NK cell killing, as indicated by similar cell viability and lysis under all conditions (53). Similarly, NKL cell cytotoxicity against the P815 mastocytoma cell line triggered by anti-CD16, anti-NKp46 and anti-2B4 (but not anti-NKG2D) was inhibited as well by anti-MHC-I antibodies when compared to an isotype antibody control (IgG2a) (54). Human blood NK cell cytotoxicity was also inhibited by anti-MHC-I antibodies when triggered by CD16, NKG2D, NKp46 and 2B4, while isotype antibody control (IgG2a) had no effect (54). Additionally, the IFN-γ secretion by NKL cells and primary NK cells was inhibited with anti-MHC-I antibodies, in contrast to incubation with isotype antibody controls (IgG2a or IgG2b) (54). Thus, most studies suggest a role for reverse MHC-I signaling in the inhibition of NK cell cytotoxicity.

Besides NK cell cytotoxicity, cell death after MHC-I triggering has been investigated. Stimulation of NK cells isolated from healthy human blood mononuclear cells with anti-CD16 antibodies led to increased cell apoptosis in comparison to unstimulated cells, which was further augmented by anti-MHC-I antibodies (51). The combined stimulation of CD16 and MHC-I in NK cells also induced TNF-α secretion in positive correlation with cell death, while no effect was seen on IFN-*γ* secretion. These results suggest that reverse MHC-I signaling plays a role in upregulating TNF-α secretion in anti-CD16-treated NK cells (51). This was further confirmed by blocking TNF-α with antibodies, which inhibited the increase in cell death induced by CD16 and MHC-I triggering, suggesting a role for TNF-α in regulating NK cell apoptosis (51). Since the functional inhibition was observed prior to the increased cell death, they were suggested to be successive. Since, in principle, adding antibodies against MHC-I molecules on NK cells could interfere with the inhibitory signals to other NK cells, resulting in reciprocal NK-to-NK cell killing, a coculture was made with two groups of NK cells treated separately with MHC-I antibodies or CD16 antibodies. This coculture had similar cell death levels to cells treated only with anti-CD16 antibodies, and decreased cell death compared to cells treated with both antibodies simultaneously. This experiment confirmed that there is an additive signaling effect when stimulating CD-16 and MHC-I on the same cell (52). Moreover, addition of anti-LFA-1 antibodies also did not change NK cell death, suggesting that LFA-1 mediated cell adhesion was not involved in the reverse MHC-I signaling process (52).

In an attempt to describe the signaling cascade downstream of MHC-I, focus was placed on NF-*κ*B. Here treatment with anti-CD16 antibodies led to induction of NF-κB activity in the nucleus, while blocking of NF-*κ*B in CD16 antibody-treated human blood NK cells induced cell death. This protective effect through CD16 stimulation was inhibited by addition of anti-MHC-I antibodies (52). Moreover, an increased expression of the NF-*κ*B inhibitor IkB and upregulation of the proapoptotic c-Jun N-terminal kinase (JNK) were observed when both antibodies against CD16 and MHC-I were used (52). Hence, it was proposed that reduction of CD16-mediated NF-*κ*B activity and increased JNK underlie the increased NK cell death after anti-MHC-I ligation.

Interestingly, in resting conditions MHC-I molecules were uniformly distributed on the surface of cells from the NKL cell line, similar to the control protein CD59 (also known as protectin), and co-localized with glycolipid-enriched membrane microdomains called lipid rafts. However, MHC-I cross-linking with primary and secondary antibodies before fixation excluded these molecules from lipid rafts in a similar way to the cell-surface transferrin receptors (CD45 and CD71), which locate outside the lipid rafts and were included as controls (53). Moreover, while MHC-I molecules were present in the immunological synapse between untreated NKL cells and the leukemia cell line K562, cross-linking with primary and secondary antibodies displaced MHC-I molecules to the opposite site of the NKL cell, away from the synapse (53). MHC-I cross-linking on NKL cells was also observed to induce intracellular tyrosine phosphorylation when compared to untreated NKL cells, although the progression of the MHC signaling pathway and immediate interaction partners are topics for further investigation (53).

An important aspect of NK cell biology is that they express a wide variety of receptors that recognize MHC-I. This raises the question whether these receptors could recognize MHC-I on the same cell (*cis* binding), and how this affects the interpretation of the literature cited in this section. A few studies have shown that *cis* binding indeed occurs (56, 57). In mouse NK cells, the inhibitory receptors Ly49A and Ly49C, which typically binds to H2-D^b,d,k^ molecules on other cells, can also associate with the allele H-2D^d^ on the same NK cell (56, 57). Although MHC-I signaling was not studied in this type of *cis* binding, it is important to highlight that Ly49A inhibitory signaling does not seem to be elicited by MHC-I molecules in *cis* (58). On the contrary, *cis* binding promotes NK cell activation as Ly49A receptors bound to MHC-I in *cis* are prevented from binding MHC-I molecules on other cells (*trans* binding) (56–59). In the case of human NK cells, *cis* binding has not been confirmed, but it has been hypothesizes to occur (60). Considering that *cis* binding is not completely well understood, it is difficult to establish to what extent the use of monoclonal antibodies against MHC-I will affect *cis* binding to NK cell receptors and its equilibrium with *trans* binding. This is mostly important in the cytotoxicity assays reviewed in this section. Furthermore, it is difficult to say how well the experimental control conditions chosen by the studies presented here (*e.g.* unstimulated cells, or stimulation with isotype controls) account for potential background *cis* binding between MHC-I and its receptors on the NK cell surface.

In conclusion, MHC molecules might be involved in reverse MHC-I signaling in triggered NK cells, mostly leading to decreased cytotoxicity and increased cell death. Possibly, this inhibition could be a way for cancer or infected cells to escape from NK cell killing *in vivo*. Furthermore, it might be a mechanism of other (healthy) immune cells to protect themselves from being attacked by NK cells. However, which specific natural ligands of MHC-I would establish such inhibitory interactions remains unknown. Potential candidates would be members of the Ly49 family in mouse, or the LILR family in human, which are expressed by different myeloid and lymphoid cells [Reviewed by (58)]. Moreover, lymphoid cells could use KIR or CD94-NKG2 receptors to communicate and regulate NK cells. In the future, consideration should be given to MHC-I itself or its downstream signaling molecules as therapeutic targets for treatment of pathologies with involvement of NK cells, such as cancer and viral infections.

### The Role of Reverse MHC-I Signaling in B Cell Proliferation and Cell Death

Cross-linking of MHC-I on human acute lymphoblastic leukemia (ALL) B cell lines and myeloid precursor cell lines by several mouse anti-MHC-I antibodies combined with a secondary rabbit-anti-mouse Ig antibody, reduced proliferation and induced apoptotic cell death, measured as DNA fragmentation. Those primary antibodies recognized epitopes in HLA-A,-B and -C or *β*_2_m and cross-linking with the secondary antibody was strictly required for apoptosis induction (61). Interestingly, MHC-I signaling also induced apoptosis in the context of mature peripheral blood B cells activated *in vitro* with an antibody against CD40, which is considered a cell rescue signal (61). In contrast, MHC-I did not have an effect in proliferation in B cells previously activated with the mitogenic bacteria *Staphylococcus aureus* or staphylococcal enterotoxin A (61). The authors suggest that MHC-I stimulation might lead to initial B cell activation, as seen previously in T cells, followed by cell death, based on the observation of an initial increase in metabolic activity in the ALL KM-3 cell line and a slow start of DNA fragmentation following MHC-I stimulation (12 h) (61) (**Table 4**). A different result was obtained by another study in which anti-MHC-I antibodies used on purified human tonsillar B cells prevented the onset of proliferation typically induced by *S. aureus*, indicating a role of MHC-I in inhibiting B cell activation (62). On the contrary, MHC-I stimulation had no effect on B cell proliferation induced by treatment with phorbol-12-myristate-13-acetate (PMA), a diacylglycerol analog that activates protein kinase C (PKC), suggesting that the inhibitory role of MHC-I targets signaling events preceding upregulation of PKC activity (62). The fact that inhibition of proliferation was not observed with F(ab)_2_ fragments of one of the antibodies used (W6/32) suggests that Fc-receptors are involved or that the Fc region inhibits interaction with another cell surface protein (62).

**Table 4:** Summary MHC-I signaling in B cells.

Cell type	MHC-I stimulation	Signaling	Cell outcome	Ref
Cell lines KM-3, HL-60 and CD40-activated B cells	Anti-MHC-I antibodies (PA2.6, W6/32, BB7.5, BB7.7 and L368) and secondary rabbit anti-mouse Ig	–	↑cell death and ↓ proliferation	(61)
Human tonsillar B cells	Anti-MHC-I antibodies (131, 4E and W6/32)	–	↓proliferation caused by *Staphylococcus aureus*	(62)
Human B lymphoma cell line Solubo	Anti-human *β*_2_m antibody from rabbit serum	–	↑ activation	(63)
Human B lymphoma cell line Loukes	Rabbit anti-human β_2_m antibody	↑protein tyrosine phosphorylation	Regulates B cell homeostasis	(64)
Human B lymphoma cell lines Solubo and Loukes	Rabbit anti-human *β*_2_m antibody and mouse anti-human HLA-ABC	↑ protein tyrosine kinases and PI-3 kinase	↓proliferation and ↑ apoptosis	(65)

Besides proliferation, short-term intracellular changes and signaling were observed upon MHC-I stimulation of B cells. Antibody-mediated cross-linking of MHC-I on human B lymphoma cells increased intracellular calcium levels, first derived from intracellular stores and later due to calcium influx (63). This increased intracellular calcium was positively correlated with the levels of MHC-I expressed by the cells (63). The early activation marker CD69 was upregulated upon cross-linking and MHC-I did not have a costimulatory effect on the anti-IgM-mediated activity of the cells (63). Finally, it was suggested that MHC-I can activate intracellular second messengers itself. For instance, upon MHC-I cross-linking, the protein tyrosine kinases p53/56^lyn^ and p72^syk^ are phosphorylated in human B lymphoma cells (64). Besides protein tyrosine kinases, serine/threonine kinases might be involved in reverse MHC-I signaling, since their inhibition with the molecule H7 reduced B cell apoptosis after MHC-I cross-linking (65).

As is the case for most of the cell types presented in this review, the physiological scenario in which reverse MHC-I signaling in B cells would be relevant remains unclear. Wallén-Öhman et al. suggested that the observed induction of apoptosis and reduction of proliferation seen upon reverse MHC-I signaling might be important for negative selection of autoreactive B cells (61). They hypothesized that, if an autoreactive B cell would target a cell bearing an MHC-I binding receptor, such as T or NK cells, the simultaneous binding of BCR-antigen and MHC-I-ligand would lead to apoptosis (61). This hypothesis was inspired by another study in which T cell precursors recognizing CTLs underwent apoptosis (66). Here, the simultaneous binding of the precursor**’**s TCR to an endogenous antigen on the CTL and of MHC-I to CD8 on the CTL induced the precursor**’**s death. This effect was not seen after T cell maturation (66). However, this hypothesis has not been tested yet for B cells.

### The Effect of Reverse MHC-I Signaling on Proliferation, Activity and Apoptosis of T Cells

Early studies found anti-MHC-I antibodies to either induce or inhibit T cell proliferation (67–71) [reviewed in (8)]. In these studies, the outcome of MHC-I stimulation depended on whether the anti-MHC-I antibody was used alone or in combination with other stimuli, and on the concentration of antibody used. On one hand, studies indicating an induction in proliferation only used anti-MHC-I antibodies (CR11-351, Q6/64, W6/32) or in combination with exogenous IL-2, PMA or antibodies against CD2. On the other hand, anti-MHC-I antibodies had an inhibitory effect when combined with stimuli that promote proliferation, such as the T cell activating lectin phytohemagglutinin (PHA)-P or antibodies against CD8 and CD3 (*e.g.* OKT3) (8, 68, 72–74). An antibody clone designated as 4–10 had a mitogenic effect and led to TCR downregulation in resting peripheral blood isolated T cells, when immobilized on a plate or on the surface of macrophages. However, when combined with immobilized anti-CD8 antibody it reduced CD8-elicited proliferation (67). Furthermore, 72 h incubation of peripheral blood T cells with the anti-MHC-I antibody W6/32 immobilized on culture plates at concentrations of 0.1 μg/ml and 1 μg/ml induced T cell proliferation, tyrosine phosphorylation and increased expression of TCR/CD3 and CD28. In contrast, very low amounts of immobilized W6/32 antibody (picograms/well) inhibited TCR/CD3-elicited proliferation. Importantly, incubation of the cells with W6/32 in solution had no effect on proliferation, even at a concentration of 40 μg/ml (68).

Induction of proliferation by Qa-2 (H2-Q7), a non-classical mouse MHC-I molecule, was compared to its human homolog HLA-G within a murine background (75). For this, resting splenic T cells were isolated from wild type mice and transgenic mice recombinantly expressing HLA-G. Stimulation of both protein complexes with monoclonal antibodies led to an induction of proliferation, but only when combined with a secondary antibody for cross-linking and stimulation with PMA (75). Both Qa-2 and HLA-G localized to lipid rafts. However, whilst Qa-2 localizes *via* a lipid glycosylphosphatidylinositol (GPI) tail, HLA-G localizes *via* its transmembrane domain. The mechanism for signaling was attributed to interactions with other lipid raft-located proteins as Qa-2 has no cytoplasmic domain and HLA-G has only a short 6-amino acid cytoplasmic tail (75, 76) (**Table 5**).

**Table 5:** Summary MHC-I signaling in T cells.

Cell type	MHC-I stimulation	Signaling	Cell outcome	Ref
Jurkat T cell line	Anti-MHC-I (W6/32) cross-linked by supernatant from W6/32-hybridoma cell cultures	↑Src, Bcl-2	In the stationary growth phase ↑ apoptosis.	(77)
Cytotoxic T cell clone K14B06	Anti-MHC-I antibodies (W6/32, HP-1F7, ME-1, anti-H2Dd)	–	↓anti-CD94 redirected target lysis	(78)
Mouse splenic T cells	Anti-HLA-G clone 87G and secondary rabbit anti-mouse IgG	–	In combination with PMA, ↑proliferation	(75)

MHC-I is also proposed to play a role in the induction of apoptosis in T cells, because anti-MHC-I antibodies induced apoptosis in resting T cells (66), activated T cells (79), and the leukemic Jurkat T cell line (63, 80–83) [reviewed in (8)]. Studies focused on Jurkat cells provided a detailed description of the early signaling cascade following stimulation with biotinylated anti-*β*_2_m antibodies crosslinked with avidin. It was shown that shortly after cross-linking, MHC-I signaling induced tyrosine phosphorylation of several proteins, including the *ζ*-chain of the TCR/CD3 complex, ZAP-70, p56^lck^ and PLC-*γ*1, followed by an increase of intracellular Ca^2+^ (81). Although similar to TCR/CD3 signaling, MHC-I stimulation led to inferior association of ZAP-70 with other molecules, a phosphorylated version of the TCR *ζ*-chain with a higher molecular weight, and weaker phosphorylation of p56^lck^ (81). Furthermore, reverse MHC-I signaling was dependent on the expression of TCR/CD3 complex on the cell surface. Overall, these results suggest that MHC-I uses the TCR/CD3 complex for signaling but creates a different pattern of protein phosphorylation compared to the pattern seen after direct stimulation of TCR/CD3. The main outcome was strong inhibition of proliferation and apoptosis induction (**Figure 2D**) (81, 84). In a similar study, crosslinking of *β*_2_m in Jurkat cells led to activation of Tyk2, but not of the other members of the JAK family Jak1, Jak 2 or Jak3. STAT3, a substrate of Tyk2, was also phosphorylated and migrated to the nucleus where it could bind to the promoter of c-fos. Expression of a dominant negative version of STAT3 interfered with apoptotic processes associated to MHC-I stimulation (80). Another study on Jurkat cells proposed that apoptosis upon MHC-I stimulation did not follow the conventional Fas/Caspase signaling pathway but rather one that includes activation of phosphoinositide 3 kinase (PI3K) followed by JNK activation (82).

Another factor determining the outcome of reverse MHC-I signaling is the cell cycle. A study reported that the effect of MHC-I ligation on apoptosis depends on the growth phase of Jurkat cells, since in exponentially growing Jurkat cells Fas- or anisomycin (SAPK/JNK activator)-induced apoptosis is prevented by MHC-I ligation. This involves Src signaling, the upregulation of anti-apoptotic protein Bcl-2 and stabilization of the mitochondrial membrane potential (77).

A potential explanation as of why MHC-I reverse signaling can have opposite effects could be the nuclear recruitment of different homo- or heterodimers from members of the NF-*κ*B/Rel family. Stimulation with antibodies against HLA-A,-B or -C leads to nuclear location of NF-*κ*B1 (p105/p50) homodimers, and formation of heterodimers by either NF-*κ*B1+RelA (p65) or NF-*κ*B1+RelB. Since NF-*κ*B1 homodimers lack transcriptional activation domains, they are considered to have an inhibitory function (85).

MHC-I reverse signaling also affects the T cell effector function. This was demonstrated in an *in vitro* experiment based on antibody-dependent cell-mediated cytotoxicity (ADCC) (78). To achieve ADCC cancer cells were coated with antibodies against the CD94/NKG2 receptor. Because the antibodies’ Fc tails bind to the Fc-receptors on the cancer cell, the paratopes of the antibody were outwardly displayed. The antibody-coated cancer cells were then mixed with CD8^+^ cytotoxic T cells, leading to CD94-mediated T cell activation and increased CD25 expression. As a result of this interaction, the T cells were prompted to kill the cancer cells (78). This ADCC triggered by CD94 ligation was shown to be independent of TCR stimulation and could be reduced by co-ligation of MHC-I molecules on the T cell. MHC-I stimulation, however, had no effect on the elevated CD25 expression, indicating that reverse MHC-I signaling affects only one branch of the CD94-elicited signaling cascade (78). Another hallmark of CD94-mediated ADCC is the reorientation of the microtubule organizing center (MTOC) of the T cell towards the synapse with the cancer cell, the same way it happens upon CD3 stimulation. MHC-I co-ligation significantly reduced CD94-mediated MTOC reorientation, explaining its inhibitory effect on target lysis (78). MHC-I stimulation did not affect cytotoxicity after CD3-mediated activation, and only had a partial effect after exposure to the T cell activating lectin phytohemagglutinin (PHA), confirming the existence of several signaling cascades underlying T cell activation and therefore different degrees of crosstalk with reverse MHC-I signaling (78).

MHC molecules can also interact with cytokine receptors at the plasma membrane. For instance, MHC-I can cluster with IL-9 receptor *α* (IL-9R*α*) and IL-2 receptor (IL-2R) in lipid rafts on human T lymphoma cells (86). Although the exact effect of MHC-I on the clustering remains unknown, several anti-MHC-I antibodies (CR11-351, Q6/64 and W6/32) block the expression of the IL-2R, as well as inhibit IL-2 secretion of T cells (73, 74, 87). Interactions within the receptor clusters may affect the signaling capability of these receptors, and although this has not yet been proven, a similar mechanism might also explain the increased insulin receptor signaling observed when MHC-I interacted with the insulin receptor on a B-lymphoblast cell line (86, 88).

Interestingly, it was observed that upon human peripheral blood lymphocyte stimulation with the T cell activating lectin PHA, the fraction of MHC-I misfolded proteins increased, as shown by immunoprecipitation with antibodies against misfolded MHC-I. The levels of misfolded MHC-I molecules peaked at the same time as T cell proliferation (30). The fact that misfolded molecules were fully glycosylated suggested that they derived from fully mature MHC-I molecules that reach the plasma membrane but lost the peptide antigen and/or the *β*_2_m subunit. It was also found that misfolded MHC-I molecules were more likely to dimerize and presented phosphorylation of the tyrosine in their cytoplasmic tail, associated with activity of the Src-kinase Lck (30). However, it is unclear whether phosphorylation is a cause or a result of the misfolding. The study also suggested that misfolded MHC-I molecules can associate with other proteins on the cell surface, such as CD8*αβ* and calreticulin, potentially regulating events related to T cell activation and its intensity. The reason for these interactions might be that misfolding and the lack of peptide unmasks amino acid motifs that are hidden in the folded form, especially in the *α*1 and *α*2 domains (29, 30).

Overall, reverse MHC-I signaling has been shown to be involved in modulation of the immune response by regulating proliferation, activity and apoptosis of T cells. These contradictory outcomes might be caused by the use of different T cells and varying stimuli across the different studies. Interestingly, there is even contradictory evidence on which MHC-I domain ensures reverse signal transduction, as some studies state that the cytoplasmic and transmembrane domains are not needed for reverse MHC-I signal transduction (89, 90), while others propose that the transmembrane region is required, as it might facilitate interaction with other surface receptors (91). The significance of the modulation of T cell function by reverse MHC-I signaling *in vivo* is not yet clear. It is thought that reduced cytotoxicity might play a role in the protection of target cells, which could be beneficial to prevent microbial dissemination from dying cells (78), but also disadvantageous if tumor cells are spared by this reduced cytotoxicity. Furthermore, it might prevent non-specific lysis of healthy cells. It is yet unknown which natural membrane-bound ligands of MHC-I are able to exert the proposed effects and if these effects are also present *in vivo*. Possibly, T cells can mutually activate each other *via* reverse MHC-I signaling, since a common ligand for MHC-I is the TCR.

## Non-Immune Cells Contribute to Inflammation by Reverse MHC-I Signal Transduction

### Epithelial Cell Proliferation and Activity Upon Reverse MHC-I Signaling

Multiple studies have shown that the development of anti-HLA antibodies by recipients of lung transplantation plays an important role in the pathogenesis of bronchiolitis obliterans syndrome (BOS). This seems to be caused by activation of airway epithelial cells by such HLA-A antibodies (92). As shown by an *in vitro* experiment modeling the transplantation environment, binding of anti-MHC-I antibody W6/32 to the KCC-266 airway epithelial cell line induced cell proliferation within 24, followed by apoptosis. The observed proliferation was accompanied by production of the growth factors platelet-derived growth factor (PDGF), heparin-binding EGF-like growth factor (HB-EGF), insulin-like growth factor 1 (IGF-1), and basic fibroblast growth factor (bFGF). These factors led to proliferation of the fibroblast cell line MRC-5, explaining the processes of tissue remodeling and formation of fibrous tissue observed in BOS (92). In a similar study, cells from the A549 lung epithelial carcinoma cell were treated with anti-HLA serum from transplantation patients who developed BOS or with the antibody W6/32. Both treatments led to induced proliferation as well as tyrosine phosphorylation of intracellular proteins. These results could be attributed to reverse signaling by MHC-I but also to signaling by MHC-II, since anti-MHC-II antibodies could be present in the serum (93) (**Table 6**).

**Table 6:** Summary MHC-I signaling in epithelial and endothelial cells.

Cell type	MHC-I stimulation	Signaling	Cell outcome	Ref
Airway epithelial cell line KCC-266	Anti-MHC-I antibodies (W6/32)	–	↑proliferation, growth factor production and apoptosis	(92)
A549 lung epithelial carcinoma cell line	Anti-HLA serum or W6/32-	Protein tyrosine phosphorylation	↑ proliferation	(93)
Primary bovine endometrial cells and bovine endometrial cell line	W6/32	↑ Fps, SHP-2 ↓ MyD88, TRAF6 and NF-κB	In combination with LPS stimulation, ↓TLR-4 signaling	(94)
Primary human aortic endothelial cells	MHC-I antibodies (IgG1 and IgG2) with a variable region of murine W6/32 and human constant region	–	↑ monocyte adhesion	(95)
Primary human aortic endothelial cells	Chimeric mouse/human pan MHC-I (IgG1), anti-HLA-A (IgG1) and human alloserum	–	Complement increased MHC-I-induced monocyte adhesion	(96)
Primary human aortic endothelial cells	Anti-MHC-I antibodies W6/32 and MEM-147	↑ mTOR, RhoA, ROCK, ERM	Regulated monocyte adhesion	(97)
Primary human aortic endothelial cells	Anti-MHC-I antibody W6/32	integrin *β*4, YAP	Increased expression of CTGF and Cyr61 → cell proliferation and migration	(98)

Similar to macrophages, MHC-I was proposed to modulate TLR signaling in epithelial cells. *In vitro* experiments showed that miRNA or siRNA knock down of MHC-I in primary bovine endometrial epithelial cells (bEECs) and a bovine endometrial cell line (BEND), led to increased secretion of the pro-inflammatory cytokines IL-1β and TNF-α after LPS stimulation (94). In accordance, MHC-I cross-linking by antibodies on bEECs decreased production of the same cytokines. Additionally, MHC-I heavy chain-silenced bEECs and BEND cells showed increased TLR4 expression, as well as increased activation of TLR4 downstream molecules, such as myeloid differentiation primary response 88 (MyD88), TRAF6 and NF-*κ*B. This was even confirmed by reduced NF-κB activity after MHC-I cross-linking (94). Interestingly, MHC-I cross-linking enhanced the interaction between MHC-I and the tyrosine kinase Fps, while Fps knock down resulted in reduced levels of SHP-2 and increased cytokine production (94). This indicates that MHC-I on endometrial cells negatively regulates the TLR4-induced inflammatory response through enhancement of the Fps-SHP-2 pathway, which might be relevant in the maternal-fetal interface (**Figure 2E**). This signaling mechanism closely resembles the one described in macrophages in section *Macrophage Activity Regulation by Reverse MHC-I Signaling* (9).

Altogether, MHC-I stimulation was suggested to cause proliferation and inhibit TLR-4 signaling in lung and endometrium epithelial cells, which could have different implications. In the lung, reverse MHC-I signaling might play a role in the development of bronchiolitis obliterans syndrome after lung transplantation. While in the endometrium, reverse MHC-I signaling might be involved in interactions between mother and fetus or even control congenital inflammatory reactions (94, 99).

### Reverse MHC-I Signaling in Endothelial Cells Contributes to Adverse Effects After Transplantation

The endothelium is a specialized form of epithelium and will therefore be separately discussed here. The role of reverse MHC-I signaling in endothelial cells has been reviewed extensively in the context of transplant vasculopathy and concomitant tissue rejection. Specifically, alloantibodies generated by the transplant recipient against MHC-I and MHC-II alloantigens present in the transplanted tissue have been linked to endothelial cell proliferation, survival and migration that can be accompanied by recruitment of inflammatory cells into the transplanted tissue (5, 7, 10, 100, 101). The organ recipient develops antibodies not only against MHC-I, but also against different types of alloantigens. However, endothelial cell lines stimulated with allosera from a rat cardiac allograft model characterized by vasculopathy and rejection *in vitro*, showed that anti-MHC-I and II antibodies were more toxic for endothelial cells, than antibodies against non-MHC antigens. This toxicity was due to complement activation upon MHC stimulation (102, 103).

In contrast to most other cell types in this review, reverse MHC-I signaling in endothelial cells has been studied in more detail and is therefore better understood. Phosphorylation of multiple signaling proteins has been observed after antibody-induced MHC-I cross-linking on endothelial cells. One of the earliest events is Ras homolog (Rho) activation immediately followed by Src, focal adhesion kinase (FAK) and paxillin activation, which together contribute to cytoskeletal remodeling and formation of focal adhesions and stress fibers (104–107). Furthermore, activation of PI3K leads to the formation of phosphatidylinositol (3,4,5)-trisphosphate (PIP3), which promotes Akt activation and its interaction with the mTOR pathway, which overall supports protein synthesis, cell survival and migration (105, 106, 108–112). Akt and mTOR activation can also promote cell survival by favoring the expression of the antiapoptotic molecules Bcl-xL and Bcl-2 (111, 113), as well as by the inhibition of the proapoptotic protein Bad (112) [reviewed in (5, 7, 10, 109)]. Notably, the effects of anti-MHC antibodies on endothelial cell signaling are independent of their Fc regions (111). Additionally, MHC-I stimulation can increase the sensitivity of the cell to growth factors by mobilizing their receptors to the plasma membrane. This is the case for the fibroblast growth factor receptor (FGFR), which promotes proliferation *via* the extracellular signal-regulated kinase (ERK) pathway (114), in a signaling cascade that is independent from one in which ERK is directly activated by MHC-I (115).

Interestingly, it was found that integrin *β*4 is an important interaction partner of MHC-I, as they coimmunoprecipitate when MHC-I is crosslinked. This interaction is likely mediated by the cytoplasmic domain of the MHC-I *α* chain, as shown by the lack of reverse signaling upon heterologous expression of *α* chains lacking the cytoplasmic domain. Knocking down integrin *β*4 led to reduced phosphorylation of FAK, Src, Akt, and ERK after MHC-I stimulation. As a consequence, endothelial cell proliferation was less when MHC-I was stimulated in the absence of integrin *β*4. Similarly, MHC-I *α* chain knock-down affected the ability of integrin *β*4 to induce migration (116, 117). A recent study found that Src signaling under MHC-I/integrin *β*4 leads to translocation of the transcriptional coactivator yes associated protein (YAP) from the cytoplasm to the nucleus. Once in the nucleus, YAP enhances the expression of connective tissue growth factor (CTGF) and cysteine-rich angiogenic inducer 61 (Cyr61), which promote endothelial cell proliferation and migration (98).

Recent studies support reverse MHC-I signaling in endothelial cells, with special focus on how it affects the recruitment and adhesion of the recipient’s monocytes. This is important because intragraft macrophages correlate with worse transplant outcomes (95–97) (**Table 6**). An *in vitro* study showed that ligation of MHC-I by antibodies for 15 min on primary human aortic endothelial cells was sufficient to stimulate the exocytosis of the cell adhesion molecule P-selectin. The display of P-selectin on endothelial cell monolayers facilitated the adhesion of two monocyte cell lines (U937 and MM6) *via* the P-selectin glycoprotein ligand-1 (PSGL-1) on their surface. Additionally, it was found that MHC-I antibodies bound to endothelial cells further favored cell-cell adhesion by binding to Fc-receptors on the monocytes with their Fc-region (95, 118). Adhesion of monocytes to anti-MHC-I antibody-treated endothelial cells was enhanced by intact complement present in the culture media compared to heat-inactivated complement. In addition, complement further promoted MHC-I-induced P-selectin expression, which was important for adhesion (96). Human IgG1 appeared to be more effective in stimulating adhesion when compared to human IgG2, in accordance with its higher affinity for Fc-receptors (95). The integrin Mac-1 (CD11b) contributed to the increase in monocyte adhesion, since neutralizing antibodies reduced adhesion (96). Importantly, there was variation between endothelial cells and monocytes from different donors, which should be considered when designing a treatment strategy for patients with transplant rejection. The *in vivo* relevance was demonstrated by an increased macrophage burden in mice with murine cardiac allografts treated with MHC-I antibodies, whereas P-selectin blockers reduced this macrophage burden (119).

Inhibition of mTOR by drugs or siRNA in endothelial cells reduced MHC-I-induced adherence of MM6 macrophage-like cells or peripheral blood mononuclear cell (PBMC)-derived monocytes (97). This reduced firm adhesion of monocytes to endothelial cells by mTOR inhibition was comparable to ICAM-1 neutralizing antibodies, suggesting that mTOR plays a role in regulating firm adhesion of monocytes to ICAM-1 on endothelial cells stimulated with MHC-I antibodies (97). Indeed, MHC-I ligation triggers ICAM-1 clustering *via* mTOR and ERM signaling (97). Moreover, mTOR activation after reverse MHC-I signaling was proposed to lead to activation of RhoA, ROCK and ezrin/radixin/moesin (ERM), as investigated with the help of small molecule inhibitors (97). Mice with a heart transplant and anti-MHC-I antibody treatment showed endothelial cell swelling and intravascular activated immune cells, which was reduced by mTOR blockade (97). Using these mice, it was also observed that anti-MHC-I antibodies increased endothelial phosphorylation of ERM, which was abolished by mTOR inhibitors (97).

In summary, reverse MHC-I signaling in endothelial cells has been linked to several cell outcomes that underlie tissue rejection, such as proliferation, migration and adhesion of immune cells like monocytes. Also, multiple mediators, such as Akt, mTOR and ERK1/2, have been associated with this signaling. Understanding these signaling pathways could facilitate the development of pharmacological interventions to treat chronic antibody-dependent tissue rejection (120). Moreover, this knowledge is not only interesting in the field of transplantation outcomes, but might also be relevant for other immune responses that require trans-endothelial migration, or diseases such as atherosclerosis in which endothelial activation and immune infiltration are involved (121).

### Proliferation and Migration of Smooth Muscle Cells After Reverse MHC-I Signaling

Reverse MHC-I signaling in smooth muscle cells has been studied in the same context of transplantation as endothelial cells (**Table 7**). Cross-linking with anti-MHC-I and a secondary antibody, but also with F(ab′)_2_ fragments, induced proliferation of human aortic smooth muscle cells (SMC) (122, 124). The same was observed with human mesenteric SMC and a human aortic SMC cell line triggered by MHC-I ligation with the monoclonal antibodies W6/32 and MEM-147 (125, 126). Moreover, proliferation was inhibited by anti-bFGF antibodies, suggesting that MHC-I stimulation triggers bFGF synthesis or uptake (122). Additionally, anti-MHC-I antibodies lead to a rapid release of stored fibroblast growth factor receptor (FGFR) to the plasma membrane and thereafter increased synthesis of FGFR (122, 123). The combination of anti-MHC-I antibody W6/32 and either TNF-α or IFN-*γ*, increased the FGFR expression even more (123). This could be explained by the finding that both inflammatory cytokines increased the expression of MHC-I (123). Besides smooth muscle cell proliferation, migration upon MHC-I ligation has also been investigated, since both cells are involved in intimal thickening of vessels during chronic rejection of a transplant. Antibody cross-linking of MHC-I on human aortic smooth muscle cells induced wound healing. This wound healing involved proliferation and migration, but also migration alone when proliferation was inhibited by mitomycin C (124). MHC-I knockdown inhibited anti-MHC-I-mediated SMC proliferation and migration (124).

**Table 7:** Summary MHC-I signaling in smooth muscle cells and myeloma cells.

Cell type	MHC-I stimulation	Signaling	Cell outcome	Ref
Human aortic smooth muscle cells	Anti-MHC-I antibodies (W6/32) and goat anti-mouse IgG	Protein tyrosine phosphorylation	↑ proliferation	(122)
Human aortic smooth muscle cells	Anti-HLA-A1 IgG and W6/32	–	↑ FGFR expression	(123)
Human aortic smooth muscle cells	Anti-MHC-I (W6/32, human anti-HLA- A24/A32 and murine anti-HLA-A2)	↑FAK, Akt and ERK1/2	↑proliferation and migration	(124)
Human mesenteric artery smooth muscle cells, human aortic SMC CRL-1999 cell line and murine aortic SMC CRL-2797	Anti-MHC-I (W6/32, anti-H-2D^b^)	↑ MT1-MMP, MMP2 and neutral sphingomyelinase-2	↑proliferation	(125)
Human mesenteric artery smooth muscle cells (primary and immortalized) and human aortic SMC CRL-1999 cell line	Anti-MHC-I (W6/32, MEM-147)	↑ SK1, S1P, and S1PR1/R3 signaling	↑proliferation	(126)
Myeloma cells	Mouse anti-human *β*_2_m (clone B2, IgG1)	↑Lyn, PLC*γ*2, JNK ↓PI3K, ERK	↑apoptosis	(127)

At the signaling level, MHC-I ligation increased the tyrosine phosphorylation of several proteins (122, 124), such as those involved in cell survival and proliferation like FAK, Akt and ERK1/2, while downregulation of MHC-I with siRNA abrogated MHC-I-induced phosphorylation (124). Additionally, decreased FAK and p-FAK after siRNA knockdown of FAK reduced the MHC-I-mediated phosphorylation of Akt and ERK1/2, indicating that their phosphorylation depends on FAK. In accordance, integrity of the actin cytoskeleton, in which FAK is involved, is important for the MHC-I-induced activation of SMC, since the phosphorylation of cellular proteins was attenuated by disruption of the cytoskeleton upon stimulation with cytochalasin D or latrunculin A (124). Overall, FAK was found to play a role in the MHC-I-mediated proliferation and migration of SMC (124).

Another major signaling pathway linked to reverse MHC-I signaling is the sphingolipid signaling pathway, related to transduction of stress signals. Early players of this pathway, such as the matrix metalloproteinase 2 (MMP2) and its target neutral sphingomyelinase-2 (nSMase2), which catalyzes the hydrolysis of sphingomyelin into ceramide, were shown to be involved in the induction of proliferation and migration by MHC-I stimulation, indicating that anti-MHC-I antibodies behave like stress-inducing agents (125). A later player of the pathway, sphingosine kinase 1 (SK1), which phosphorylates sphingosine to sphingosine-1-phosphate (S1P), and the SP1 receptors S1PR1/R3 were also required for the induction of proliferation and migration by MHC-I stimulation, as observed when using several antibodies, inhibitors and silencing by siRNA (126). Altogether, it can be proposed that after MHC-I stimulation, MMP2 is activated and acts on nSMase2 to stimulate ceramide generation (100). Ceramide can then be converted to S1P, which activates Akt and MAPK/ERK pathways and thereby promotes proliferation (100, 126). However, the signaling cascades are probably more complex than this proposed model, as for instance S1P was suggested to have a role as extracellular mediator (126).

Immunodeficient SCID/beige mice grafted with human mesenteric segments and injected with W6/32 anti-MHC-I antibody showed vascular abnormalities (vasculopathy) in the transplant, which was characterized by intimal hyperplasia. These vascular abnormalities were not observed in untreated mice or mice treated with irrelevant antibody (125, 126). This suggests that also *in vivo* reverse MHC-I signaling plays a role in smooth muscle cell proliferation and migration, of which the former was confirmed by an increase in proliferating cell nuclear antigen (PCNA) (125, 126). Moreover, increased intimal MMP2 expression was observed, while MMP and nSMase2 inhibitors (partly) prevented the increased PCNA expression and intimal thickening (125). Besides, PCNA labeling and intimal thickening were reduced by anti-S1P antibodies, supporting the notion that W6/32 stimulates SMC proliferation *via* S1P. However, it is uncertain if these outcomes are caused by direct reverse MHC-I signaling or are the result of inflammatory cytokine production (126).

In conclusion, MHC-I signaling can induce SMC proliferation and migration, but the complete molecular pathway remains to be elucidated. The induction of proliferation and migration of smooth muscle cells by MHC-I-stimulation might contribute to transplant vasculopathy, a hallmark of chronic rejection after transplantation (122, 124). Several reviews have addressed the role of MHC-I antibodies in SMC activation, also in relation to transplant outcomes (7, 100, 101, 128). However, anti-MHC-I antibody-specific effects need to be validated in human cardiac transplants showing vasculopathy.

## Reverse MHC-I Signal Transduction Results in Cancer Cell Apoptosis

### The Potential of *β*_2_m Stimulation in Inducing Myeloma Cell Death

To study the potential of monoclonal antibodies in cancer treatment, the effect of anti-β_2_m antibodies on myeloma cells was first investigated *in vitro*. Anti-*β*_2_m antibodies, but surprisingly not the antibody W6/32 which targets a conformational epitope in the MHC-I *α*-chain, induced apoptosis of human myeloma cell lines and human primary myeloma cells. This apoptotic effect was not counteracted by myeloma pro-survival cytokines such as IL-6 and insulin-like growth factor-I (127). In contrast, non-cancer cells such as PBMCs, were not killed, which could be due to the higher expression of *β*_2_m and HLA-ABC on myeloma cells (127). Knockdown of *β*_2_m expression on myeloma cells by siRNA induced resistance to the apoptosis (127). Upon anti-*β*_2_m treatment, MHC-I was internalized and caspase-9, -3, -7 and poly (ADP-ribose) polymerase (PARP) were activated in myeloma cells (127). At the same time, the proapoptotic proteins Bad and Bax were upregulated while expression levels of antiapoptotic Bcl-2 and Bcl-xL did not change (127). In combination with an increase in cytosolic cytochrome c, this indicates increased permeability of mitochondria (127). Moreover, levels of phosphorylated JNK increased, phosphorylated Akt and ERK decreased and partial JNK inhibition almost completely abrogated myeloma cell apoptosis (127). After anti-*β*_2_m treatment, MHC-I molecules were recruited to lipid rafts and disruption of lipid raft structure by methyl-*β*-cyclodextrin treatment abrogated apoptosis (127). Immunoprecipitation showed that Lyn and PLCγ2 associated with MHC-I, which were both phosphorylated after antibody treatment (127). Altogether, this suggests that due to anti-*β*_2_m antibodies, MHC-I locates to lipid rafts, activates Lyn and PLC*γ*2, which activate JNK and inhibit Akt and ERK pathways and finally induce myeloma cell apoptosis. In mice, anti-*β*_2_m injections reduced tumor volumes and improved survival, whereas no other tissues were damaged, and pJNK, cleavage of caspase-9 and caspase-3 and myeloma apoptosis were induced (127). Further evidence on the potential of anti-*β*_2_m antibodies as therapeutic agent for multiple myeloma has been reviewed extensively elsewhere (11, 129).

Altogether, it is likely that *β*_2_m is involved in the regulation of cell death in myeloma cells and it might be interesting to study the role of MHC-I (components) in other tumor cell types. Drug targeting *β*_2_m might be a potential treatment for myeloma patients, but it should be confirmed that there are no effects on other cells than myeloma cells as suggested by Yang et al. (127). However, the ability of tumor cells to reduce their surface MHC-I expression should be taken into account (130). It could also be useful to study if and to which extent cell death of tumor cells by ligation of MHC-I or *β*_2_m occurs in cancer patients. Finally, as also mentioned in section *Macrophage Activity Regulation by Reverse MHC-I Signaling*, the effects triggered by anti-*β*_2_m antibodies might not be exclusively related to reverse MHC-I signaling, since other heterodimers such as CD1 and the neonatal Fc-receptor (FcRn) are also formed by *β*_2_m. Hence, the interpretation of the data requires caution.

## Concluding Remarks

Here we summarized available evidence of reverse MHC-I signaling. Reverse MHC-I signaling has been observed in multiple immune and non-immune cells *in vitro* as well as *in vivo*. MHC-I signaling has been shown to affect cell apoptosis, activation (*e.g.* growth factor production), proliferation, cytotoxicity and migration in many cell types, including immune cells, epithelial cells and tumor cells (3, 5, 6). Because of these broad actions, reverse MHC-I signaling is relevant to several fields, including research into viral and bacterial infections (6, 9), transplantation outcomes (9) and malignancies (11). One difficulty in the study of reverse MHC-I signaling has been separating it from the canonical roles of MHC-I in antigen presentation for T cell activation and NK cell suppression. This is technically difficult because deletion of MHC-I would interfere with both these functions and would therefore also impair the formation of the immunological synapse. As a consequence, most studies have used monoclonal antibodies to stimulate cell surface MHC-I, leaving the physiological relevance *in vivo* to be elucidated. Similarly, the *in vivo* ligands that induce reverse MHC-I signaling are often unknown. One exception is observed in transplantation biology, where anti-MHC-I antibodies are produced by the tissue recipient.

Identifying the MHC-I domain important for reverse signaling has been controversial. Wagner et al. concluded that the cytoplasmic domain of MHC-I is not needed for signal transduction, whereas the transmembrane part is (91). In contrast, others found that neither the cytoplasmic nor the transmembrane region of MHC-I are needed for reverse MHC-I signaling, and suggested that the extracellular domain interacts with other cell surface molecules to ensure signal transduction (89, 90). More recently, cytoplasmic tyrosine phosphorylation of MHC-I was observed and phosphorylated MHC-I was co-immunoprecipitated with Fps, suggesting a role of the cytoplasmic domain in reverse signaling (9). Similarly, others suggest that the first event following MHC-I ligation is an interaction of MHC-I with intracellular Lyn and PLCy2 in tumor cells (127) or association with other surface receptors on T cells (89). Additionally, the presence of putative PDZ (PSD95/disc large/zonula occludens-1) ligand motifs in the cytoplasmic region of MHC-I suggests potential interactions with PDZ domain-containing proteins *in vivo* (26). This is further supported by the fact that these PDZ ligand-like regions are conserved across species and seem to be under positive selective pressure. Moreover, *in vitro* experiments showing that MHC-I cytoplasmic domains can bind to immobilized PDZ domain peptides, are the starting point to study MHC-I downstream signaling *via* its cytoplasmic domain (26). Nevertheless, it is possible that the cytoplasmic, transmembrane and extracellular domains of MHC-I are all important for signal transduction, depending on the cell type and stimulus.

Although the exact pathways following MHC-I ligation are not yet completely known for all cell types, several mechanisms have been suggested (**Figure 2**). The emerging picture shows commonalities between different cell types, where signaling molecules/pathways triggered by reverse MHC-I signaling are shared: Fps and SHP-2 as early events in macrophages and epithelial cells (9, 94); the involvement of STAT proteins in macrophages and T-cells (6, 80); JNK in NK cells and tumor cells (52, 127); Akt and ERK1/2 signaling in endothelial cells (106, 108, 110–113, 115), smooth muscle cells (124, 126), and myeloma cells (127); and an inhibition or activation of NF-κB in macrophages, NK cells, T cells and epithelial cells (9, 52, 85, 94) (**Tables 1**–**7**). However, as not all of these signaling pathways are studied in all cell types, it might well be that the reverse MHC-I signaling mechanism is actually quite similar in different cell types. Also, the signaling pathway and outcome might depend on the cell subtype (*e.g.* resting blood T cells *vs*. Jurkat T cells), differentiation state, type of MHC-I stimulus, and co-stimulation (42, 61). A recurrent pattern among different cell types is the crosstalk between reverse MHC-I signaling and signaling pathways initiated by for instance TLR, Fc receptors, integrins and growth factor receptors (9, 31, 94, 115–117). Such a cross-talk with other signaling pathways would position MHC-I molecules as major modulators of immunological physiology and homeostasis.

Interestingly, similar outcomes and signaling pathways to MHC-I stimulation have been described after stimulation of MHC-II molecules in professional antigen-presenting cells, such as B cells and DCs [reviewed in (131)], as well as in other immune cells, like T cells and monocytes (132–134). In B cells, reverse MHC-II signaling was suggested to play a role in B cell activation, proliferation, differentiation or apoptosis after B cell-T cell interaction [reviewed in (135–137)], and also killing of malignant B cells (135, 138). Similar to MHC-I, both cytoplasmic tail-dependent and -independent mechanisms have been proposed for reversed MHC-II signaling (135, 136). Another similarity to reverse MHC-I signaling is the location of MHC-II signaling molecules in lipid rafts. While stimulated MHC-II molecules present in lipid rafts of antigen-presenting cells can signal *via* tyrosine kinases to ensure activation, maturation or proliferation, the MHC-II molecules that are relocated to non-raft regions can signal *via* protein kinase C to induce cell death (131). Likewise, in murine B cells the way in which the *α*- and *β*-chains of MHC-II interact with each other creates different conformers, 10% of which locate preferentially in lipid rafts where they interact with the B cell receptor (BCR) partner CD79 to induce tyrosine kinase activity (139). Other intracellular reverse MHC-II signaling events similar to reverse MHC-I signaling are for example tyrosine phosphorylation and activation of Src, AKT, ERK and JNK (135). Also, the transmembrane adaptor protein SCIMP was shown to be tyrosine phosphorylated after MHC-II stimulation serving as scaffold for downstream signaling pathways (140). A potential role of SCIMP or a similar scaffold protein in MHC-I signaling could explain the connection observed to tyrosine kinases. T cell studies showed that similar to MHC-I, MHC-II ligation also inhibits the activation cascades elicited by CD3 stimulation with OKT3. This inhibition was evidenced by reduced proliferation and reduced expression of the cytokines IL-1β, IL-6 and IL-2, and of the IL-2 receptor when compared to T cells treated with only OKT3 (132–134). While reverse MHC-I signaling has been poorly studied in DCs, studies on MHC-II in these cells showed induced maturation and apoptosis. Monocyte-derived DCs underwent maturation when stimulated with a pan-HLA-DR epitope (PADRE) when bound to particles, in a process involving MAP kinase, p72^syk^ and NF-κB (141). Moreover, stimulation of HLA-DR molecules in plasmacytoid DCs lead to apoptosis but only when already mature (142). These commonalities strengthen the idea of a general regulatory role for MHC molecules on immune and non-immune cells, beyond their antigen presenting function.

Looking forward, the field of reverse MHC-I signaling has great potential for basic and applied research. Despite that it is increasingly clear that reverse MHC-I signaling has major effects on many cell types, our knowledge of this process is still in its infancy. The main limitation in the field is our poor knowledge of the ligands that bind MHC-I *in vivo*. Potential candidates include the members of the human KIR family and their murine functional homologs in the Ly49 family, members of the LILR and PIR family, as well as members of the CD94-NKG2 family. The fact that the expression of some of these receptors is not limited to NK and T cells, but extends to myeloid cells [reviewed in (58)], opens the possibility that multiple types of cell-cell interactions could be shaped by reverse MHC-I signaling. Moreover, the search for MHC-I ligands could be broaden to other non-immune receptors, as shown by a study on the role of MHC-I in neuronal development. This study proposes that MHC-I and the insulin receptor are expressed in different sets of neurons and can bind to each other in *trans*. This interaction allows MHC-I to downregulate the synapse-promoting role of the insulin receptor, leading to a reduced density of synapses during brain development. However, this study did not look at potential signaling downstream of MHC-I (143). Another important challenge in the field is to establish what the first signaling event after MHC-I stimulation is, as this is required for a complete understanding of reverse MHC-I signaling. In many of the studies cited in this review, this first molecular interaction was elusive and only the downstream signaling pathways were confirmed. Confirming functional relevance of the putative PDZ domains of MHC-I (26) and identifying their interaction partners (*e.g.* by mass spectrometry) could help to identify these early signaling events. Moreover, the reported recruitment of MHC-I molecules to lipid rafts raises the question whether reverse MHC-I signaling is regulated by the clustering and segregation of MHC-I with other receptors (53, 75, 76, 86, 127). It would also be important to study whether the signaling processes reported previously *via* the different MHC-I domains (cytoplasmic, transmembrane, extracellular or *β*_2_m) can actually coexist, expanding the modulatory capacity of MHC-I. In order to address these questions, approaches used in the past for the study of TCR and BCR signaling could come in handy, using for example purified ligand candidates (as mentioned above) tethered to supported lipid bilayers (144, 145). Furthermore, it would be important to assess the contribution of reverse MHC-I signaling cellular outcomes, such as proliferation and apoptosis, to *in vivo* physiological and pathological processes. Finally, future efforts could focus on the identification of potential therapeutic approaches to target reverse MHC-I signaling for managing tissue transplantation [see for example (120)], and treatment of viral and bacterial infections, as well as malignancies. If successful, this new knowledge would establish that MHC molecules are not only passive displays of antigenic peptides, but are also genuine immune receptors that modulate the intracellular signaling within the APC.

## Author Contributions

EM, LM and NR wrote the manuscript and GB participated in discussion and reviewed/edited the manuscript. All authors contributed to the article and approved the submitted version.

## Funding

NR is funded by a Long-Term Fellowship from the European Molecular Biology Organization (EMBO-LTF, ALTF 232-2016) and a Veni grant from the NWO Talent Scheme (016.Veni.171.097). GB is funded by a Young Investigator Grant from the Human Frontier Science Program (HFSP; RGY0080/2018), and a Vidi grant from the Netherlands Organization for Scientific Research (NWO-ALW VIDI 864.14.001). GB has received funding from the European Research Council (ERC) under the European Union’s Horizon 2020 research and innovation programme (grant agreement No. 862137).

## Conflict of Interest

The authors declare that the research was conducted in the absence of any commercial or financial relationships that could be construed as a potential conflict of interest.
